# Supplementary material for: Existing function in primary visual cortex is not perturbed by new skill acquisition of a non-matched sensory task
Source: Nat Commun. 2022 Jun 25;13:3638. doi: 10.1038/s41467-022-31440-y (PMC9233699; doi:10.1038/s41467-022-31440-y)
Supplement: Supplementary file 3 — Description of Additional Supplementary Files [file 41467_2022_31440_MOESM3_ESM.pdf]

Description of Additional Supplementary Files

File Name: Supplementary Movie 1
Description: Example of control signal threshold crossings during direct neuron selection process.

File Name: Supplementary Movie 2
Description: Example BCI trial, session #1.

File Name: Supplementary Movie 3
Description: Example BCI trial, LP session.

File Name: Supplementary Movie 4
Description: Example BCI trial, the last session.

File Name: Supplementary Movie 5
Description: Locomotion tracking.

File Name: Supplementary Movie 6
Description: Example mouse performing the visual discrimination task.
