# Supplementary material for: Existing function in primary visual cortex is not perturbed by new skill acquisition of a non-matched sensory task
Source: Nat Commun. 2022 Jun 25;13:3638. doi: 10.1038/s41467-022-31440-y (PMC9233699; doi:10.1038/s41467-022-31440-y)
Supplement: Supplementary file 10 — Reporting Summary [file 41467_2022_31440_MOESM10_ESM.pdf]

nature portfolio

Corresponding author(s): Sandra Kuhlman

Last updated by author(s): May 6, 2022

Reporting Summary

Nature Portfolio wishes to improve the reproducibility of the work that we publish. This form provides structure for consistency and transparency in reporting. For further information on Nature Portfolio policies, see our Editorial Policies and the Editorial Policy Checklist.

Statistics

For all statistical analyses, confirm that the following items are present in the figure legend, table legend, main text, or Methods section.

n/a	Confirmed	
☐	☒	The exact sample size (*n*) for each experimental group/condition, given as a discrete number and unit of measurement
☐	☒	A statement on whether measurements were taken from distinct samples or whether the same sample was measured repeatedly
☐	☒	The statistical test(s) used AND whether they are one- or two-sided<br>*Only common tests should be described solely by name; describe more complex techniques in the Methods section.*
☒	☐	A description of all covariates tested
☐	☒	A description of any assumptions or corrections, such as tests of normality and adjustment for multiple comparisons
☐	☒	A full description of the statistical parameters including central tendency (e.g. means) or other basic estimates (e.g. regression coefficient) AND variation (e.g. standard deviation) or associated estimates of uncertainty (e.g. confidence intervals)
☐	☒	For null hypothesis testing, the test statistic (e.g. *F*, *t*, *r*) with confidence intervals, effect sizes, degrees of freedom and *P* value noted<br>*Give P values as exact values whenever suitable.*
☒	☐	For Bayesian analysis, information on the choice of priors and Markov chain Monte Carlo settings
☒	☐	For hierarchical and complex designs, identification of the appropriate level for tests and full reporting of outcomes
☒	☐	Estimates of effect sizes (e.g. Cohen's *d*, Pearson's *r*), indicating how they were calculated

Our web collection on statistics for biologists contains articles on many of the points above.

Software and code

Policy information about availability of computer code

Data collection	ScanBox version 1.13 (Neurolabware), Matlab version 2021a.
Data analysis	Normality was tested using IBM SPSS software (version 26), as well for Chi Square testing. All other statistical tests and analysis were done in the Matlab coding environment. Acquired calcium imaging signal was analyzed using Suite2p version 1.0.0.0.

For manuscripts utilizing custom algorithms or software that are central to the research but not yet described in published literature, software must be made available to editors and reviewers. We strongly encourage code deposition in a community repository (e.g. GitHub). See the Nature Portfolio guidelines for submitting code & software for further information.

Data

Policy information about availability of data

All manuscripts must include a data availability statement. This statement should provide the following information, where applicable:
- Accession codes, unique identifiers, or web links for publicly available datasets
- A description of any restrictions on data availability
- For clinical datasets or third party data, please ensure that the statement adheres to our policy

The data generated in this study have been deposited in the GIN database, available at https://gin.g-node.org/bjeon/V1_BCI.git.

Field-specific reporting

Please select the one below that is the best fit for your research. If you are not sure, read the appropriate sections before making your selection.

☒ Life sciences ☐ Behavioural & social sciences ☐ Ecological, evolutionary & environmental sciences

For a reference copy of the document with all sections, see nature.com/documents/nr-reporting-summary-flat.pdf

Life sciences study design

All studies must disclose on these points even when the disclosure is negative.

Sample size	No prior calculation was done because before the present study there was no data that could be used to estimate the magnitude of the response variance before and after training, which would be required to calculate the number of points needed to achieve statistical power. The data collected demonstrate statistical significance at the 0.05 level.
Data exclusions	One mouse was removed from the study due do technical issues as described in the main text.
Replication	The materials to conduct these experiments are widely available. Our analysis is expected to be replicable given we provided the datasets required to do so. Prior findings were not attempted to be replicated.
Randomization	Male and female subjects were randomly selected.
Blinding	Investigators were not blind to allocation into experimental groups in the case repeated measures designed was used (all animals experienced the same treatment).

Reporting for specific materials, systems and methods

We require information from authors about some types of materials, experimental systems and methods used in many studies. Here, indicate whether each material, system or method listed is relevant to your study. If you are not sure if a list item applies to your research, read the appropriate section before selecting a response.

Materials & experimental systems

n/a	Involved in the study	
☒	☐	Antibodies
☒	☐	Eukaryotic cell lines
☒	☐	Palaeontology and archaeology
☐	☒	Animals and other organisms
☒	☐	Human research participants
☒	☐	Clinical data
☒	☐	Dual use research of concern

Methods

n/a	Involved in the study	
☒	☐	ChIP-seq
☒	☐	Flow cytometry
☒	☐	MRI-based neuroimaging

Animals and other organisms

Policy information about studies involving animals; ARRIVE guidelines recommended for reporting animal research

Laboratory animals	Adult laboratory mice (Mus musculus) >41 days old of both sexes (as labeled in manuscript) were used. The ambient temperature range was, 68-75 degrees F, and the humidity range was 18-65%. To express the calcium indicator GCaMP6f selectively in excitatory neurons, either homozygous Emx1cre mice (Jackson Laboratories, stock number 005628) or homozygous SLC17a7cre mice (Jackson Laboratories, stock number 023527) were crossed with homozygous Ai93/heterozygous Camk2a-tTA mice (Jackson Laboratories, stock number 024108). Experimental mice were heterozygous for all three alleles.
Wild animals	The study did not involve wild animals.
Field-collected samples	The study did not involve samples collected form the field.
Ethics oversight	All experimental procedures were compliant with the guidelines established by the Institutional Animal Care and Use Committee of Carnegie Mellon University and the National Institutes of Health, and all experimental protocols were approved by the Institutional Animal Care and Use Committee of Carnegie Mellon University.

Note that full information on the approval of the study protocol must also be provided in the manuscript.
